# Lessons learnt during COVID-19: making sense of Australian and Swedish university lecturers’ experience

**DOI:** 10.1186/s41239-023-00395-5

**Published:** 2023-04-28

**Authors:** Kristina Turner, Siobhan O’Brien, Helena Wallström, Katarina Samuelsson, Sirkka-Liisa Marjatta Uusimäki

**Affiliations:** 1 Swinburne University of Technology, Melbourne, Australia; 2 La Trobe University, Melbourne, Australia; 3 University of Gothenburg, Göteborg, Sweden

**Keywords:** Sensemaking, COVID-19, University lecturer, Wellbeing, Digital technologies

## Abstract

This article reports on a study analysing changes in the use of digital technologies and working from home during the COVID-19 crisis and the impact of these changes on the wellbeing of five female university lecturers from Australia and Sweden. Applying collaborative autoethnographical methods, this study employed Weick’s sensemaking framework to explore how the academics made sense of these sudden changes. The Positive emotion, Engagement, Relationships, Meaning, and Accomplishment (PERMA) wellbeing framework was also employed to explore the effect of these changes on the academics’ wellbeing. Findings from the reflective narratives show that after the initial experiences of stress, each university lecturer was able to adapt and navigate the online teaching environment during the pandemic. However, the time constraints in preparing and adapting to online teaching, and working from home, were experienced by some of the university lecturers as highly stressful and isolating which impacted their sense of wellbeing. Even so, working from home was recognized as a positive experience, providing time for research, hobbies, and time with family. This study addresses a gap in current knowledge by examining the impact of the sudden transition to online teaching and learning had on academic wellbeing as conceptualised through the PERMA framework. In addition, by applying Weick’s sensemaking framework, this study provides a unique perspective around how academics made sense of the sudden switch to online teaching and learning during COVID-19.

## Introduction

COVID-19 has both transformed and impacted university lecturers’ professional and personal lives. Initially, the COVID-19 circumstances were thought to be simply a pause to normal life (Almpanis & Joseph-Richard, 2022). Remote teaching was considered a temporary shift of instructional delivery due to crisis circumstances and expected to return to the original format once the crisis abated (Hodges et al., 2020). However, this shift was far from temporary, with the COVID-19 pandemic enduring through the 2020 and 2021 academic years (Almpanis & Joseph-Richard, 2022). While lockdowns eased in some parts of the world, simultaneously in other countries they were re-imposed due to ‘second wave’ infections (Ellis et al., 2020).

The circumstances of ‘lockdowns’ in Australia included the closure of schools and universities. Leaders in higher education institutions across Australia responded with an almost overnight application of digital communication methods that supported university lecturers working from home and students learning remotely for sustained periods. As scheduled the classes were quickly transformed into online learning, and university lecturers and students were required to connect in a virtual space. Whilst, leaders in the Swedish universities followed closely the recommendations set by the Swedish Public Health Authority and, while there were immediate lockdowns of all universities and colleges at the onset of COVID-19, the lockdown did not apply to the compulsory schooling sector or society at large. Despite the immediacy of the closure of classes in the middle of the second term, all universities were able to provide all university students with a seamless transition to online learning (Author et al. 2022, blinded for review).

In undertaking this work, we explored the literature and reviewed papers that considered the facilitation of teaching and learning during the pandemic. We found that Almpanis and Joseph-Richard (2022) had effectively recounted the implementation of a variety of educational technologies including Blackboard, Moodle, Canvas, Adobe, and web-conferencing tools such as Zoom and MS Teams. The authors also considered teaching methods which incorporated synchronous lectures, live ‘Question and Answer’ sessions, online breakout room discussions, text-based online discussions, live-chat, asynchronous lectures, and pre-recorded commentary of PowerPoint presentations. Ellis et al. (2020) also revealed experiences that aligned to our own in that throughout the transition to online teaching and learning university lecturers engaged with ‘Known Unknowns’ and ‘Unknown Unknowns’ which required them to stabilise the situation and rethink practices within the complex context (p. 564).

Impositions to moving to remote learning, including the enforced use of digital technologies for teaching, and learning practices were time-constrained (Almpanis & Joseph-Richard, 2022; Barnes et al., 2021). With little time to prepare or adapt to the revised pedagogical techniques required for successful online teaching and learning, even with provisions for technical and pedagogical support from institutions, many university lecturers felt unprepared and overwhelmed at the complex task (Almpanis & Joseph-Richard, 2022; Barnes et al., 2021; Joseph et al., 2021). For example, hours of work were required to modify teaching materials and assessments that had been designed for face-to-face classroom teaching (Almpanis & Joseph-Richard, 2022). Interacting with students online was also quite different for academics. Students were more likely to request one-to-one Zoom meetings and assignment extensions (Joseph & Trinick, 2021). Unexpected interruptions also occurred during live sessions with ‘family members or pets appearing in the Zoom Class’ (Joseph & Trinick, 2021, p. 217). The increased workload saw university lecturers from around the world running on empty (Ellis et al., 2020, p. 564).

Positive outcomes from the transition to remote contexts included university lecturers increased digital literacy skills through their applied understanding and competency of digital tools (Barnes et al., 2021). Interestingly, Almpanis and Joseph-Richard (2022) noted a ‘shift from content creation to interactive online facilitation’ (p. 386). Whilst, Joseph and Trinick (2021) revealed how university lecturers formed informal communities of practice, gathering online to talk about the challenges they were experiencing. University lecturers shared ideas to improve practice to support the delivery of interactive and engaging sessions (Joseph & Trinick, 2021). In addition, the challenges in making meaningful connections with students in an online environment cued a renewed focus for an inclusive student-centred strengths-based pedagogical approach (Ní Dhuinn & Garland, 2022).

Examples of studies that have examined university lecturers’ use of digital technologies during the COVID-19 pandemic include a study by Badiozaman (2021) which examined Malaysian higher education teachers’ experiences of transitioning to online teaching and learning. This study revealed that the transition to online teaching presented an unprecedentedly complex scenario for higher education teachers and required tremendous resilience (Badiozaman, 2021). Whilst in a study of South African university lecturers, Vandeyar (2021) revealed that in response to the move to online teaching during the COVID-19 pandemic, lecturers prioritised an ethic of care, compassion and social justice for students in their teaching practice.

However, these studies did not apply a sensemaking framework (Weick et al., 2005) to examine how academics made sense of the sudden shift to online teaching and learning during the COVID-19 pandemic, nor did they explore the impact of this change on academic wellbeing. Weick et al.’s (2005) sensemaking framework is discussed in the next section.

## Sensemaking

Sensemaking is an ongoing process in which people seek to ‘make sense’ of events that are surprising, confusing or, in contrast with the ‘normal’ situation (Ganon-Shilon & Schechter, 2017). Individuals’ or groups’ construction of the meaning of such events is mediated by their prior knowledge, experiences, beliefs, and values, and is embedded in the social context within which they work (Ganon-Shilon & Schechter, 2017; Weick et al., 2005). Sensemaking is triggered by events whose meaning is uncertain or ambiguous, leading individuals or groups to question what is going on and what they should do next (Ganon-Shilon & Schechter, 2017; Weick et al., 2005) for example, the COVID-19 crisis. The process of sensemaking rationalizes what has occurred and brings order to the disorder (Ganon-Shilon & Schechter, 2017). Weick et al. (2005) asserts that disruptions in routine processes require individuals to make sense of what is occurring now and to consider what should be done next.

Previous research in the field of sensemaking addresses how sense is made of crises after they happen (Maitli & Sonenshein, 2010), and the behaviour of individuals and groups in complex situations (Weick, 1993). Sensemaking occurs on both subconscious and conscious levels. At the sub-conscious level, it assists with organising the understanding of what has happened and the development of plausible explanations and solutions (Weick, 1993). However, when under threat humans do not always make the right or most effective choices and Weick (1993) proposes that it is our humanness that plays the major part in a chaotic scenario.

Sensemaking has previously been applied within the context of higher education (see for example Zerquera & Ziskin, 2020) and was determined to be the most appropriate framework for this study to explore how academics made sense of the sudden switch to online teaching and learning during COVID-19. The process of sensemaking can be applied when there is an unexpected instance, or unplanned event (Weick, 1995). The COVID-19 pandemic crisis was such an event and required people to make sense of large amounts of information (Christianson & Barton, 2021). Without prior experience working within the conditions of a pandemic, we became university lecturers in periods of chaos facing conditions of threat. Through a sensemaking lens we examined the processes we undertook to ‘make sense’ of the impact of the COVID-19 pandemic on our work life and sense of wellbeing. As a form of analysis, sensemaking provided us with a process to interpret and reinterpret these events which when put into context helped us to make sense of what has happened to us (Weick, 2007).

Applying collaborative autoethnographical methods, this study employed Weick’s sensemaking framework to explore how the academics made sense of these sudden changes. The Positive emotion, Engagement, Relationships, Meaning, and Accomplishment (PERMA) wellbeing framework was also employed to explore the effect of these changes on the academics’ wellbeing. The PERMA framework is discussed in the next section.

## PERMA wellbeing

This study applied the Positive emotion, Engagement, Relationships, Meaning, and Accomplishment (PERMA) wellbeing framework (Seligman, 2012) to examine the impact on university lecturers’ wellbeing when they used digital technologies for remote teaching during the COVID-19 pandemic. ‘Positive emotion’ is the subjective measure of happiness and life satisfaction, ‘engagement’ is the subjective measure of being absorbed in a task and ‘positive relationships’ are relationships with others that support wellbeing (Seligman, 2012). ‘Meaning’ is the subjective experience of belonging to or serving something which you believe is bigger than yourself and ‘accomplishment’ refers to experiencing achievement or success (Seligman, 2012).

Previously, the PERMA wellbeing framework has been used to examine wellbeing in higher education. For example, Silva Munar et al. (2020) explored organizational happiness and PERMA wellbeing in higher education institutions in Chile, Colombia and Spain. Whilst Zewude and Hercz (2022) examined PERMA wellbeing in an Ethiopian higher education setting. In addition, Oades et al. (2011) proposed a framework for building ‘positive universities’ based on the PERMA framework similarly, Turner (2022) examined the relationship between ‘servant leadership theory’ and the PERMA wellbeing framework, suggesting servant leadership and servant teaching as a potential model for improving wellbeing in higher education academics.

However, a review of current literature revealed a gap in understandings of the effect which the sudden switch to the use of digital technologies for online teaching and learning during the COVID-19 pandemic had on university lecturers’ wellbeing. Whilst there are a few studies examining individual elements of academics’ wellbeing during COVID-19 pandemic, for example: Naylor and Nyanjom (2021) studied Australian lecturers’ emotions, Huang et al., (2022) examined Chinese lecturers’ engagement, Hardman et al. (2022) examined South African lecturers’ relationships with colleagues and Dinu et al. (2021) reported on English academics’ accomplishment. There is a dearth in literature examining the effect the switch to remote teaching and learning during the COVID-19 pandemic had on university lecturers’ wellbeing as conceptualised through the PERMA framework.

This study addresses a gap in current knowledge by examining the impact of the sudden transition to online teaching and learning had on academic wellbeing as conceptualised through the PERMA framework. In addition, by applying Weick et al.’s (2005) sensemaking framework, this study provides a unique perspective around how academics made sense of the sudden switch to online teaching and learning during COVID-19. The power of sensemaking is that it provided insight into the challenges and changes the lecturers were experiencing, such as adapting to the new work-from-home environment, engaging, and learning the use of new digital technologies as well as teaching skills for a new online environment. The COVID-19 pandemic created and unpredictable situation which involved unprecedented challenges (Kim et al., 2022) such as lecturers’ usual work practices were disrupted with the transitioning from on-campus teaching and learning to working from home. The pandemic environment was complex, novel, and rapidly changing (Christianson & Barton, 2021), for lecturers this offered opportunities for growth and learning (Kim et al., 2022) and demanded that they ‘make sense’ of the changing nature of their work and how the changes impacted their sense of wellbeing.

## Method

This study employed a qualitative collaborative autoethnography research method. Collaborative autoethnography is “a qualitative research method that is simultaneously collaborative, autobiographical, and ethnographic” (Chang et al., 2012, pp.17), which draws on the researchers’ lived experiences, combined with existing literature, to develop new knowledge (Fourie, 2021). Collaborative autoethnography was determined to be the most appropriate method for this study as it has previously been applied with Weick et al.’s (2005) sensemaking framework to examine how employees make sense of uncertainty in a work-related context (see for example: Brommel, 2017; Samuels et al., 2021; Vickers, 2007). Collaborative autoethnography typically uses a small sample size which allows for in-depth exploration of the experiences and perspectives of the individual participants (Creswell, 2012). Thus, this study with five participants, has a valid sample size. As per autoethnographic research methods (see for example, Chang et al., 2012; Creswell, 2012), data was collected through written reflections and conversations.

The five participants in this autoethnographic study are all employed as academic staff in universities. Kristina is a senior lecturer in primary teaching and course director of the Master of Teaching (Primary) course at a university in Melbourne, Australia. She teaches undergraduate pre-service teachers in Mathematics, Social and Emotional Learning and Arts initial teacher education units. Siobhan is a senior lecturer and professional practice coordinator at a university in Melbourne, Australia. She works with undergraduate pre-service teachers in the use of learning innovations including virtual reality simulations and social media. Helena is a senior lecturer in educational science at a university in Gothenburg, Sweden. She teaches advanced level units in the master programs in the Department of Education and Special Education. Katarina is a senior lecturer at a university in Gothenburg, Sweden. She teaches in undergraduate and advanced level teacher educations and master programs in the Department of Education and Special Education. Liisa is an associate professor at a university in Gothenburg, Sweden. She has extensive experience with Scandinavian pedagogical and educational leadership approaches and teaches both undergraduate and postgraduate students in project management, fostering international collaborations, mentoring, leading teams, promoting inclusion, academic well-being, and digital literacy.

### Participants and data

The five female participants of the study are the authors. Ethics approval was not required by the relevant university ethics committees for a self-study. Each author has experience (4–15 years) working as a university lecturer as well as four with a background in classroom teaching. Two authors are employed at a major university in Melbourne, Australia, and three are working at a university in Gothenburg, Sweden. The participating Swedish and Australian authors had not met previously, the Chair of the Department of Education from a university in Melbourne provided the introduction between the partners.

The below reflective questions (Table 1) were developed by a Swedish lecturer (Liisa) and relate to exploring university lecturers’ experiences and making sense of the changes to online teaching and learning and the impact of the COVID-19 crisis on their sense of wellbeing. These questions were developed from a variety of informal Swedish academic staff survey questions sent at the beginning of the pandemic to different departments at the relevant university.

**Table 1:** Reflective questions

Questions to the Swedish and Australian colleagues
What have been the positives of working online during the pandemic? (Include personal challenges working from home etc.)
What have been the main challenges working online during the pandemic? (Include personal challenges working from home, family, health, and wellbeing, etc.)
How confident are you about your digital literacy skills?
What pedagogical principles work when teaching online?
How do you activate students learning and how do you know when students are actively engaged in learning?
How do you understand the differences between online, hybrid, and face-to-face teaching and learning? Do you have a preference?
How confident are you about your student’s digital literacy skills?

Liisa forwarded the questions via email to each of the lecturers in Sweden and Australia. Once each of the lecturers had provided and shared their reflective email responses with each other, a mutual Zoom meeting was held where, each of the lecturers was provided time, to comment, question, share, and/or seek further clarification on any of the reflective email responses they had received and edit or modify questions. This iterative process formed the model for all online meetings. The reflective responses formed the data for the study. This paper reports on a portion of these findings related to changes in the use of digital technologies and working from home during the COVID-19 crisis and the impact of the changes on the participant’s sense of wellbeing.

### Analysis

Qualitative methodological integrity (Levitt et al., 2018) was addressed through two of the researchers independently conducting manual thematic analysis (Creswell, 2012) of the five participants’ data. First, each of the participant’s responses were colour coded into meaningful chunks which allowed the initial themes to be identified. At this stage online discussions were held with all five researchers to resolve differences of opinions and agree on the core central themes which emerged from the data. We placed the sections of text under the following thematic headings: (i) digital technologies (ii) wellbeing (including PERMA framework sub themes of: emotions, engagement, relationships, meaning and accomplishment), (iii) regulating professional work, (iv) nurturing collegiality and (v) mastering online technology. This article relates to themes (i) and (ii). Themes (iii), (iv) and (v) are addressed in a separate article.

Then reading and re-reading these lived experiences the same two researchers employed a sensemaking framework (Weick et al., 2005) to recognize, explain and interpret our experiences, identifying similarities, differences, and interconnections, between our lived experiences. Again, at this stage, online discussions were held with all five researchers to resolve differences of opinions and agree on the sensemaking themes which emerged from the data. Through this processes, three sensemaking themes emerged: (i) collaboration, (ii) wellbeing, and (iii) meaning making.

## Findings

In this section, we present the findings ascertained from the thematic analysis under the thematic headings (i) Digital Technologies and (ii) Wellbeing.

### Digital technologies

The transition to online teaching and learning occurred rapidly both at Australian and Swedish universities. The sudden switch from face-to-face teaching to working from home and learning using new digital technologies and pedagogical skills for online teaching and learning was especially stressful for the Swedish lecturers whose experience, in main, had been teaching face-to-face. In making sense of this rapid change, both Katarina and Helena turned to their colleagues for support. For instance, Katarina (Sweden) experienced stress relating to a lack of knowledge of digital technologies and online teaching, yet by turning to her colleagues she found how ‘*almost overnight we became a problem-solving team, there were many things and problems to solve such as we needed to change a large on-campus three-day course to a digital course.’*

While there was a need to learn new digital technologies and online teaching skills all participants shared a basic sense of competency in their digital literacy skills and a level of self-confidence and adaptability to online teaching and learning. Siobhan (Australia) found that professional development was easier to access at her university:

*‘Due to the professional development sessions running online I attend more sessions than I would have in person. Our university has had a recent shift to Adobe Creative Campus with sessions run by our learning and transformations unit. I used Adobe web page instead of PowerPoint for my presentations. I have also become quite confident with Zoom, Teams, and our Canvas interfaces that we have used for online learning.’*

Kristina (Australia) also felt confident with her level of digital literacy, and where there were skills that she did not have, she felt confident enough in her ability to learn them:

‘*I felt very supported by the university Information Technology department as they have always been very helpful when I have called them with a problem. I have also used deficits in my digital technology skills as teaching moments with my pre-service teachers to demonstrate that it is OK not to know everything and to ask for help when you need it.’*

The Swedish perspective was similar with digital technology professional learning opportunities. However, the upskilling process was not without its challenges. Katarina (Sweden) explained how she learned to manage Zoom; *‘This was a new tool for me. I learnt to use break-out rooms to interact with my students*.’ Although Katarina (Sweden) experienced university support before teaching and learning sessions, there was less university support available during teaching and learning sessions. To illustrate, ‘*when I had a lecture for four hundred students, I was the only one there from the university*.’ Helena (Sweden) attended some digital technology professional development sessions but found support through her colleagues and the internet: *‘much of the teaching was managed by myself with help from the internet, for example how to record my lectures.’*

Both Swedish and Australian participants reflected that teaching online offered new affordances and constraints for teaching. For example, Katarina (Sweden) observed that ‘*some of the positives were that many students who would never raise their voice in a big lecture could now ask questions *via* the chat but, this can be hard to follow while you’re teaching*.’ Whilst, Siobhan (Australia) introduced applications like Padlet to allow the students to interact however, she expressed surprise at the number of students who also needed to learn how to use new technologies:

‘*I am always surprised at what they do not know. I tend to assume they know more than me or will be more enthusiastic to learn new skills. We have asked them to learn Adobe portfolio for their assessment—some are not so keen. We also ask them to use Instagram during their placement—many of them are not so into social media but do see the advantage of a professional account once they get started. They are also required to participate in a virtual classroom simulation using MURSION as a part of an assessment task. It acts as a practice before they go into schools. This technology has had mixed responses.’*

Many participants reported that students’ use of cameras was both an affordance and a constraint. Helena (Sweden) noted that: ‘*in smaller groups of students like twenty persons, I have asked the participants to switch on their cameras. But when it has been about 100–150 students, it has not been possible to recognize all of them on camera, so I worked with small groups in breakout rooms instead.’* Whilst Siobhan (Australia) expressed a belief that academics could not expect students to turn their cameras on:

*‘I do not ask them to turn cameras on. I do encourage them though—I tell them that I feel like a radio DJ. It is much better when they do because the visual changes the dynamics. I teach a placement class and most students had a remote placement—when they were doing these, they become more comfortable with cameras on. They realised that as teachers they needed to.’*

Participants revealed that the sudden switch to online teaching and learning was a catalyst for change to their teaching and learning practice and indicated an intention to carry these changes forward in their post-pandemic teaching and learning practice. For example, Kristina (Australia) reported how her teaching practice improved as she provided students with more opportunities to communicate with her by including more ‘*interactive checkpoints’* which required students to pause and reflect on their learning. In addition, she made changes to the unit content which meant there was more direct teaching of important content. ‘*I will carry this forward into my post-pandemic teaching practice*.’

Considering their experience with online teaching during the pandemic, we asked participants to consider their personal preference between online, hybrid, and face-to-face teaching and learning. Kristina (Australia) noted that her preference is for a hybrid model involving online resources and readings, including short asynchronous ‘lecture’ style videos which are supported with weekly face-to-face teaching and learning classes:

‘*This model is most supportive of a variety of student learning styles. It offers students an opportunity to engage with learning materials in a time, place and manner that suits them, with the ability to review the materials as often as required. In addition, it also offers the benefits of face-to-face learning including, interacting with, and learning from peers, experiencing a sense of connectedness, and belonging to the university and the opportunity to establish positive and supportive relationships with academic staff.’*

However, Katarina (Sweden) explained that she still preferred face-to-face teaching: *‘I miss the informal chat between the participants. As far as I have understood it many student conversations are running in parallel forums such as Facebook.’* She described the value of the informal chat in creating a sense of belonging and friendships, and in the sharing of ideas. Katarina (Sweden), questioned if it is possible to foster and support these elements effectively in an online forum: ‘*I have tried in different ways such as ‘organized’ cafés before a lecture starts but to me this is not comparable to face to face informal chats.’*

To date, various published studies examining academic experiences of digital technologies for remote teaching during COVID-19 show similar and aligned insights to our experiences. For instance, Ní Dhuinn and Garland, (2022) noted how the re-defined delivery included both synchronous and asynchronous modes were enacted in a short space of time and had more emphasis on providing an opportunity for student engagement. Cutri et al. (2020) also found that for effective transition access to professional learning and digital technology support was necessary as well as being ‘open to change’. At times, digital technology issues caused participants in this study to experience feelings of distress and added to the heightened feelings of isolation. Participants also reported a propensity to become overwhelmed at university expectations and the amount of work required to rapidly switch to online teaching and learning. This experience was reiterated in other studies (see for example: Cutri et al., 2020), showing that this was a universal phenomenon.

### Wellbeing and digital technologies

#### Emotions

Both Swedish and Australian participants reported experiencing positive and negative emotions in relation to their use of digital technologies during COVID-19. Joy was an example of a positive emotion that all participants reported experiencing. For example, Helena (Sweden) expressed joy in her newly learned technological skills which were enabling her to work effectively at online teaching and learning. Liisa (Sweden) expressed ‘*Highly enjoying digital technologies’* and conveyed feeling grateful that she was able to support her students to continue with their studies throughout COVID. Similarly, Kristina (Australia) communicated joy in her ability to support her students as they learned to navigate online teaching and learning, whilst Siobhan (Australia) enjoyed the affordances that digital technologies offered enabling her to continue to connect with her students throughout the pandemic. So too, Katarina (Sweden) was pleasantly surprised ‘*How fast we adapted and changed our ways of teaching and working*.

However, participants also reported feeling some negative emotions in relation to their use of digital technologies during COVID. Swedish academics Helena, Liisa, and Katarina all expressed feelings of frustration and stress triggered by technological difficulties such as microphones or the internet not working which interrupted their teaching sessions. To illustrate, from Katarina (Sweden): ‘*The stress was awful when being responsible for an online lecture for four hundred students, not knowing if the programs / camera / microphone/sound would work.’* Australian academics Siobhan and Kristina reported stress around adjusting to new work routines. For example, from Siobhan, *‘Feeling like I was in a never-ending loop of long work hours with far too much time spent sitting with little or no activity.*

In contrast to Regan et al.’s (2012) United States pre-COVID study of teacher educators’ experience of online teaching, in which participants reported more negative than positive emotions, participants in this study expressed positive emotions in relation to their use of digital technologies during COVID-19. They were grateful for the affordances offered to be able to continue teaching and connecting with their students during the COVID-19 pandemic. The COVID-19 pandemic context and enforced constraints on teaching practice resulting from the pandemic, in addition to several years of technological advancement in the field of online teaching (2012 to 2020/2021) accounts for the difference in the higher education academics’ emotional response to online teaching in these two studies. Whilst participants in this study experienced some technology-related challenges or ruminated over the potential for technology-related challenges, they expressed confidence that they would receive support if they required it. This is in line with Naylor and Nyanjom’s (2021) descriptions of futuristic educators who experience satisfaction, self-efficacy, and perseverance in transitioning to online teaching.

#### Engagement

All participants reported in relation to their use of digital technologies during COVID-19 that they found their work to be interesting and engaging. For example, Liisa (Sweden) reflects that she may have accepted too many projects during this time as she found them all so interesting. Likewise, Siobhan (Australia) expressed that she found the opportunity to be involved in solving the many technological challenges which occurred because of the COVID-19 lockdown, such as pre-service teachers needing to do professional placement online, to be very interesting. So too, Kristina (Australia) indicated that she found when working online that the days seemed to go by very fast as she was often completely absorbed in her work due to the minimal interruptions in her home office.

There is a small number of studies that have previously examined university teacher’s engagement in online teaching environments. In one such study conducted in China during the COVID pandemic, Huang et al., (2022), concluded that online teaching may lead to university lecturers feeling less engaged with teaching. Further, and concerningly, Huang et al. (2022), also revealed that university lecturers’ work engagement was positively related to their self-efficacy and teaching satisfaction, and negatively related to their emotional exhaustion. In contrast, the results of this current study indicated that university lecturers’ engagement was stimulated by their use of digital technologies in an online learning environment. Whilst further research is required to better understand the reasons for these contradictory findings, it could be argued, on the basis of Huang et al.’s (2022) findings, that the increased engagement with teaching experienced by participants in this study, may have potentially also resulted in improvements in their self-efficacy and satisfaction, and a reduction in their emotional exhaustion.

#### Relationships

All participants reported that in relation to the use of digital technologies during COVID-19 that they often felt disconnected from their colleagues. Helena (Sweden) stated, *‘I often felt disconnected and lonely. I find it much easier to communicate in real life. Not often there is room for spontaneous, relational, and more reflective conversations online’* Similarly, Kristina (Australia) stated, ‘*I often felt disconnected from colleagues, it was hard to connect in online meetings especially when all the cameras were off*.’ Likewise, Katarina (Sweden) reflected, ‘*I really missed the spontaneous chats, socializing and problem-solving that occurs in a workplace. There were many colleagues with whom I had no contact for a very long time, and I did not meet many of those who got employed during the pandemic’.*

To date, very few studies examining academics’ relationships with their colleagues in the context of online teaching and learning environments gave been located. Whilst all participants in this study reported that during online teaching, they often felt disconnected from their colleagues, previous studies by Dinu et al. (2021) and Hardman et al. (2022) reported that academic staff experienced a greater sense of collegiality and developed more positive, caring relationships with colleagues during the period of online teaching. Further research needs to be conducted to better understand these discrepancies and to ascertain strategies for mitigating academics’ sense of loneliness and isolation during online teaching.

#### Meaning

Participants from the two countries reported differing responses about the impact which teaching online had on how valuable they perceived their work to be. To illustrate, in Melbourne Australia where the COVID-19 pandemic enforced lockdown was in place for almost two years (the longest lockdown in the world), Kristina and Siobhan both reported that they found providing online wellbeing support to students who were struggling with isolation and online learning to be very valuable and meaningful. Whereas in Sweden where there was no enforced lockdown, Liisa, Katarina and Helena reported that teaching online was no more or less valuable than teaching face-to-face, although they all agreed that teaching in any context was a valuable and rewarding occupation.

Previous research suggests that when teachers find meaning in their work it is supportive of their subjective wellbeing (see, for example, Dik et al., 2009). Thus, participants’ perception of their work as meaningful would be supportive of their wellbeing. Interestingly, the Australian participants in this study described supporting student wellbeing as meaningful, in contrast to the findings of Hardman et al. (2022) in which participants described supporting student wellbeing as keeping them from attending to meaningful work such as research. Further research is required to better understand these conflicting findings. In addition, there is a dearth of research in the field of university lecturer perception of meaning at work and the authors recommend that more research is conducted in this field, especially in the context of online teaching and learning environments.

#### Accomplishment

Most academics reported that working online during the COVID-19 pandemic supported them in achieving their goals during this period. For example, Siobhan (Australia) explained that she used the time in which she would normally have been commuting to and from work to complete her Ph.D. However, Katarina (Sweden) cautions, ‘*I accomplished my goals, but they came at a price. Previously it was easier to keep my home a place for family and relaxation. Now I was always at work, not having to spend time on a bus to work thus came at a cost.’* Helena’s (Sweden) experience as a newcomer in academia was less conducive to achieving goals, *‘I was a newcomer in academia when the pandemic started and one of my goals was to get to know my new workplace and colleagues, which has been hard since the room for spontaneous conversations are limited online. Also, I have tried to write some research articles, and this has been a tough activity without support from colleagues.’*

Similar to Dinu et al. (2021) and Cronin (2022), most of the participants in this study reported a perception of increased productivity whilst working from home during the COVID-19 pandemic. However, some of them also acknowledged that this increased productivity came at the cost of difficulty in maintaining a work-life balance. So, whilst the perception of increased accomplishment at work is supportive of participants’ subjective wellbeing, the challenges of maintaining a work-life balance may impact negatively on participants’ subjective wellbeing. More research is needed to better understand the interplay of these factors on higher education teachers’ wellbeing whilst teaching online.

In this section, we presented the findings ascertained from the thematic analysis under the thematic headings (i) Digital Technologies and (ii) Wellbeing. The following section presents a sensemaking discussion which explores how the participants made sense of their experiences of switching to online teaching and learning during the COVID-19 pandemic.

## Sensemaking discussion

In this section, we present the insights ascertained from the sensemaking analysis and discuss these under the thematic headings: (i) Collaboration, (ii) Wellbeing, and (iii) Meaning making.

### Collaboration

The COVID-19 pandemic caused a sudden change in circumstances which interrupted university lecturers’ usual teaching and learning practices and required them to ‘re-enact’ (Maitli & Sonenshein, 2010) their work environments. As the university lecturers in this study experienced doubts about the switch to online teaching and learning, they turned to their colleagues for support, collaboration, and sensemaking in ‘re-enacting’ their teaching and learning practices. Doubt is an essential component of adaptive sensemaking (Maitli & Sonenshein, 2010; Weick, 2007) as it drives and energizes individuals to generate possibilities, try them out, modifies, transform, or abandon them (Locke et al., 2008, p. 908).

This study confirmed previous findings of sensemaking as a process of social construction (Weick et al., 2005), in which individuals draw on distributed knowledge within their organisation to develop understanding about what is happening and what should be done about it (Christianson & Barton, 2021; Maitli & Sonenshein, 2010). Through collaboration, individuals make key decisions that determine and shape the reforms which they bring in, emphasize, or filter out (Ganon-Shilon et al., 2017). High levels of collaboration were evident in participants’ responses. For example, from Katarina (Sweden) ‘*almost overnight we were a problem-solving team ….’*

Christianson and Barton (2021) assert that in sensemaking, individuals act their way into knowing, and then generate explanations for these actions retrospectively (Weick, 1995). However, in this study, it appeared that the participants actions were guided by well thought out explanations. For example, introducing Padlet and Zoom breakout rooms to allow the students to interact. This discrepancy between the findings of this study and previous research requires further research to better understand.

### Wellbeing

The application of digital technologies for remote teaching during COVID-19 had an impact on academic wellbeing. Applying the PERMA wellbeing framework, this study reveals that participants expressed positive emotions in relation to their use of digital technologies during COVID-19 (for example: joy, interest, and engagement in work). However, participants also reported feeling some negative emotions (for example: frustration, stress, and a sense of disconnection from colleagues).

Emotion plays an important role in the sensemaking process (Ganon-Shilon & Schechter, 2017). Weick (1993) asserts that change and crises are inherently ambiguous events that typically generate confusion, fear, and anxiety for those involved. Intense, negative emotions typically found in crisis are especially likely to impede sensemaking through consuming cognitive capacity (Maitli & Sonenshein, 2010). However, positive emotions can shape sensemaking in ways that can avert crisis and enable constructive change (Maitli & Sonenshein, 2010) by broadening individuals’ scope of attention, creativity, cognitive capacity, and resilience (Fredrickson et al., 2003). In fact, optimistic sensemaking after a crisis can have a powerful, beneficial effect on individuals enabling recovery and replenishment (Maitli & Sonenshein, 2010). Consistent with previous sensemaking research, participants’ sensemaking was affected both positively and negatively by the emotions they experienced during the COVID-19 crisis.

### Meaning making

Participants in this study adjusted quickly to online remote teaching requirements, the impetus being the shared priority to connect with their students and maintain student engagement in learning. Participants’ responses differed in relation to the impact which teaching online had on how valuable and meaningful they perceived their work to be. Kristina and Siobhan (Australia) both reported that they found providing online wellbeing support to students who were struggling with isolation and online learning to be very valuable and meaningful. Whereas in Sweden, Liisa, Katarina and Helena reported that teaching online was no more or less valuable than teaching face-to-face (although they all agreed that teaching in any context was a valuable and rewarding occupation). Interestingly, the university lecturer participants in this study reported that working online during the COVID-19 pandemic supported them in achieving their goals during this period, although they cautioned that this came at a cost to work-life balance.

Sensemaking literature proposes that individuals retrospectively turn their lived experiences into cognitive maps thus developing plausible meanings which rationalize what they are doing (Ganon-Shilon & Schechter, 2017; Weick et al., 2005). This was a period of reflexive change in which our interactions and understanding of the world shifted dramatically and sensemaking helped us to construct meaning where there was no prior knowledge or experience (Weick, 1995). This process supports individuals to make sense of what is going on and informs how they engage in the situation (Ganon-Shilon & Schechter, 2017). This was evidenced in Australian academics’ sensemaking with their actions (providing online wellbeing support to students) preceding their rationalization of the action (as valuable and meaningful). During crises, people tend to notice the things they can affect (Maitli et al., 2010) thus, Australian academics in their unique lived experience of the longest period of lockdown in the world, noticed that they could affect student wellbeing.

## Limitations

The authors acknowledge the limitations of this study. Firstly, the aim of qualitative research is not to produce generalizable findings, but to explore the experiences of a small sample of individuals (Creswell, 2012). Therefore, further research applying a variety of methodologies, including longitudinal studies, are recommended to explore how university lecturers working in different contexts make sense of sudden change, and the impact of that change on their wellbeing. In addition, in qualitative data analysis, including collaborative autoethnography methods as in this study, the researchers must apply rigorous methods to minimize researcher subjectivity (Creswell, 2012). In this study, qualitative methodological integrity was addressed through two of the researchers independently conducting thematic analysis of the data and then online discussions with all five researchers followed to resolve differences of opinions and agree on the core central themes which emerged from the data.

A further limitation to this study is that it does not include insights from students. Looking at the student experience of learning in these formats would have offered a way for us to triangulate and perhaps even counterbalance our responses of this period.

## Conclusion

The impact of COVID-19 on higher education has been diverse and profound worldwide and varies from institution to institution. The beginning of the pandemic was experienced by the lecturers in this study as a time of stress and confusion with a sense of isolation and loneliness mainly because of the transition from on-campus teaching and learning, to entirely online. The disruption meant making sense of, and adapting to, new digital technologies, digital terms, pedagogical skills, and working from home. The blurring of being ‘constantly’ available online, the hours spent rewriting and preparing lecture material for online whilst figuring out plans B and C in case of internet outages or other unforeseen happenings, impacted on lecturers’ sense of wellbeing. With each lecturer becoming more confident both with the digital technologies and working from home there was a sense of joy and accomplishment, which meant recognizing the positives of online teaching and learning and working from home, such as more time with family, more time for writing and especially, saving on travel time to and from the university. Thus, the shift to online teaching and learning during the pandemic has shown the undeniable powerful effects of digital technologies on higher education, effects that will continue to grow in importance in the future. However, this is not without challenges such as addressing social isolation and disconnections for lecturers and students.

Employing Weick et al.’s (2005) sensemaking framework for analysis of the data in this study revealed participants’ intuitive and natural processes in response to unknown and changing situations during the COVID-19 pandemic. During the COVID-19 disruptions, participants turned to their colleagues for support, collaboration, and to make sense of the sudden, enforced changes to their teaching and learning practices. The participants’ ability to make sense of this change was affected both positively and negatively by the emotions they experienced during this time. However, propelled by the priority of connecting with their students and maintaining student engagement during this time, they quickly adjusted to online remote teaching requirements.

The pandemic illuminates the importance of studying sensemaking in ways that are more attentive to the complex and dynamic environments in which sensemaking takes place and that encompass longer spans of time. For example, studying sensemaking trajectories will enrich our understanding of the factors that shape the unfolding of sensemaking over time. (Christianson et al., 2021). Sensemaking can be especially useful in educational policy implementation since educators’ act based on what has meaning for them. More specifically, educators make sense of external policies, which in turn affects the change in their practices. Reform implementation is strongly influenced by educators’ understanding of it, in addition to the larger policy environment in which the reform is implemented. Policymakers, therefore, should allow educators a wider space to make sense of the reform to be implemented, considering the specific needs of local contexts.

More research is needed studying sensemaking in extreme contexts to allow sensemaking scholars to better understand sensemaking (Christianson & Barton, 2021). Sensemaking may inform higher education online teaching and learning policy and practice, and academic wellbeing policy and practice, as universities move toward a blended model of practice in the post-pandemic landscape.

## Data Availability

The datasets used and/or analysed during the current study are available from the corresponding author on reasonable request.
